# A Budding Relationship: Bacterial Extracellular Vesicles in the Microbiota-Gut-Brain Axis

**DOI:** 10.3390/ijms21238899

**Published:** 2020-11-24

**Authors:** Sandor Haas-Neill, Paul Forsythe

**Affiliations:** 1 McMaster Brain-Body Institute, The Research Institute of St. Joseph’s Hamilton, Hamilton, ON L8N 4A6, Canada; haasneis@mcmaster.ca; 2 Firestone Institute for Respiratory Health, St. Joseph’s Healthcare and Department of Medicine, McMaster University, Hamilton, ON L8N 4A6, Canada

**Keywords:** microbiome, microvesicles, probiotics

## Abstract

The discovery of the microbiota-gut-brain axis has revolutionized our understanding of systemic influences on brain function and may lead to novel therapeutic approaches to neurodevelopmental and mood disorders. A parallel revolution has occurred in the field of intercellular communication, with the realization that endosomes, and other extracellular vesicles, rival the endocrine system as regulators of distant tissues. These two paradigms shifting developments come together in recent observations that bacterial membrane vesicles contribute to inter-kingdom signaling and may be an integral component of gut microbe communication with the brain. In this short review we address the current understanding of the biogenesis of bacterial membrane vesicles and the roles they play in the survival of microbes and in intra and inter-kingdom communication. We identify recent observations indicating that bacterial membrane vesicles, particularly those derived from probiotic organisms, regulate brain function. We discuss mechanisms by which bacterial membrane vesicles may influence the brain including interaction with the peripheral nervous system, and modulation of immune activity. We also review evidence suggesting that, unlike the parent organism, gut bacteria derived membrane vesicles are able to deliver cargo, including neurotransmitters, directly to the central nervous system and may thus constitute key components of the microbiota-gut-brain axis.

## 1. Introduction

The microbiota–gut–brain axis refers to wide-ranging interactions between the gut microbiota and the central nervous system which involve endocrine, immune, and neural signaling pathways [1]. Over the past decade there has been growing interest in the contribution of the microbiota–gut–brain axis to neurodevelopment and mental health.

One of the first indications that the gut microbiota can alter brain function was the observation by Sudo et al. [2] that germ-free mice—which lack microbiota—have a hyperactive HPA axis, with higher levels of stress-associated hormones, corticosterone and adrenocorticotropic hormone (ACTH) released following restraint stress, when compared to mice with conventional microbiota [2]. There are now numerous studies indicating that the gut microbiota plays a role in development of the CNS and modulates systems associated with stress responses, anxiety [3,4,5] and memory [1]. Qualitative differences in gut microbiota composition has been associated with mood and behavioral disorders while exposure to specific nonpathogenic bacteria can modulate brain chemistry and behavior in adult animals [3] and modify depressive and anxiety-like symptoms in human subjects [6,7]. Exposure to certain commensal bacteria in early life can attenuate the effects of stressors on CNS development [8,9,10,11]. While studies strongly support the existence of a microbiota–gut–brain axis there is limited understanding regarding how signals from specific organisms, or groups of organisms, are transmitted from the gut to the brain. However, there is evidence that a number of pathways can contribute to the ability of gut microbes to modulate CNS function:

(i) Gut microbes can engage “hardwired” neural signaling between gut and brain through interaction with the enteric nervous system (ENS) and vagus nerve [12,13,14,15,16,17]. The vagus nerve is the major afferent pathway between the gut and the CNS [18] and a number of studies suggest that integrity of vagal afferents is critical to the effects of specific gut microbes on brain function and behavior [3,16,19,20,21]. Vagal afferent fibers are present in all layers of the digestive tract wall, however, these fibers do not cross the intestinal epithelial layer, thus luminal microbiota cannot interact with them directly [22]. However, vagal chemoreceptors may be activated directly by bacterial derived substances, such as short chain fatty acids, that can be transported across the epithelial barrier to the portal circulation, or by paracrine factors, such as serotonin (5-HT), cholecystokinin (CCK), glucagon-like peptide-1 (GLP-1), and peptide YY (PYY), released from enteroendocrine cells following stimulation of microbial pattern recognition receptors on their luminal side [23,24,25,26]. There is also evidence that intrinsic primary afferent neurons (IPAN) of the ENS, which make up the majority of sensory fibers innervating the intestinal mucosa, may act as the initial neural detectors of microbial signals and in turn modulate vagal activity via intramural synaptic transmission [27].

(ii) Gut microbes can modulate the activity of peripheral and central immune cells leading to altered behavior and stress responses [4,28,29,30,31,32,33]. The peripheral immune system is recognized as a major modulator of mood and behavior [34]. Gut microbiota interaction with gut epithelial cells can modulate IgA secretion, influencing dendritic cell maturation and phenotype [31]. Dendritic cells and microfold cells in the gut can also directly uptake microbiota, altering antigen presentation and consequently, T cell responses [31]. Immunosuppressive regulatory T cells altered indirectly induced by the presence of specific gut bacteria have been shown to elicit antidepressant behavioral changes in mice [33]. Changes in the composition of gut microbiota can also alter the number of activated monocytes in peripheral circulation [35] and migration of these cells to the brain has been shown to regulate hippocampal neurogenesis and behavioral responses to stress inflammatory cells such as neutrophils, which can have their activation inhibited via short chain fatty acids, and alternatively via inflammatory monocytes bound to microbial ligands [35,36,37].

(iii) Gut microbes can activate endocrine responses of the host and signal the brain via the circulation [38,39]. In addition to stimulating vagal afferent fibers, the hormones released by enteroendocrine cells following microbial interaction (CCK, PYY, 5-HT, GLP-1) also enter the circulation and regulate many metabolic processes unrelated and in addition to brain function and or behavior [38]. Commensal bacteria also indirectly regulate release of HPA hormones such as cortisol, modifying the stress response and anxiety-like behavior [2,40]. This control is achieved through the microbial secretion of short chain fatty acids which are able to diffuse past the intestinal epithelium to induce epigenetic change, as well as bind to G protein coupled receptors: GPR43, GPR41, and GPR109A on the surface of cells to transduce downstream hormone release [41,42].

(iv) Gut microbes can themselves release metabolites, including neurotransmitters, that travel through the circulation to the central nervous system (CNS) [1,43,44]. Neurotransmitters, including serotonin, dopamine, noradrenaline, and GABA, are both indirectly regulated by the presence of certain gut bacteria, and are produced by certain bacteria [45,46,47]. Neurotransmitters and their precursors, hormone-like metabolites, and short chain fatty acids are transported or diffuse across the epithelial barrier and into the blood, in which they are carried throughout the body including to the brain where they act on respective receptors to modulate neuronal and microglial function [41,42,43].

There is limited knowledge of mechanisms underlying the ability of microbes to engage each of these arms of the microbiota–gut–brain axis, but evidence is accumulating that systems, originally identified as facilitating communication between bacteria, are involved in inter-kingdom signaling and play a key role in maintaining the relationship between microbiota and host. In particular, vesicles derived from either Gram positive or Gram negative bacteria, collectively referred to in this review as bacterial membrane vesicles (MV), are emerging as potential key components of microbe–host signaling.

The study of bacterial MV as mediators of inter-kingdom communication is a relatively new field and there has been little focus on their potential contribution to the gut–brain axis. In this review, we discuss what is currently known about the MV released by the microbiota and the ways in which they may influence behavior and cognition. Evidence suggests that not only can MV interact with all the same peripheral pathways influencing behavior as the parent bacteria, they can also travel to the brain and interact with the CNS directly. Thus, bacterial MV may play an important role in modulating behavior and controlling the course of mood disorders including anxiety and depression.

### Bacterial MV

While once considered merely as a mechanism of cellular waste disposal, extracellular vesicles (EV) are now recognized as an important intercellular communication system, evolutionarily conserved throughout archea, bacteria, and eukaryotes [48].

Bacterial membrane vesicles are lipid bilayer capsules shed from the membranes of bacteria and range from 20 to 400 nm in diameter [49]. Produced by pathogenic and commensal organisms, bacterial MV are perpetually generated in the gastro-intestinal tract by the 10^13^–10^14^ bacteria residing therein [12,44]. Both Gram negative and Gram positive bacteria shed MV [50,51]. Gram negative MV are shed from the bacterial outer membrane and are thus made of a lipopolysaccharride membrane with a periplasm center [52]. The Gram positive MV membrane composition has not been fully elucidated but lipoteichoic acid is expressed on their surface and Resch et al. [53] reported an abundance of phosphatidylglycerol and a dearth of cardiolipin in relation to the parent bacterial cell membrane. There are several hypotheses of the biogenesis of Gram positive MV but none are universally accepted as of writing—broadly however, cell wall modification is thought to be involved [54]. Cell wall modification has been shown to occur in *Bacillus subtilis* with the recruitment of endolysin which cleaves holes in the peptidoglycan and allows the cell membrane underneath to bud through them [49,55]. There is evidence that *Staphylococcus aureus* also uses endolysin and potentially another class of peptidoglycan cleaving enzyme, autolysins, to allow for membrane budding [56]. Autolysins have been found inside *S. aureus* derived MV, and knockdown of genes for the specific autolysin, Sle1 has shown it to aid in MV release, particularly around junctions between dividing cells [57]. This evidence suggests that puncture of the cell wall is an important step in Gram positive MV release [54,57].

**Figure 1:**
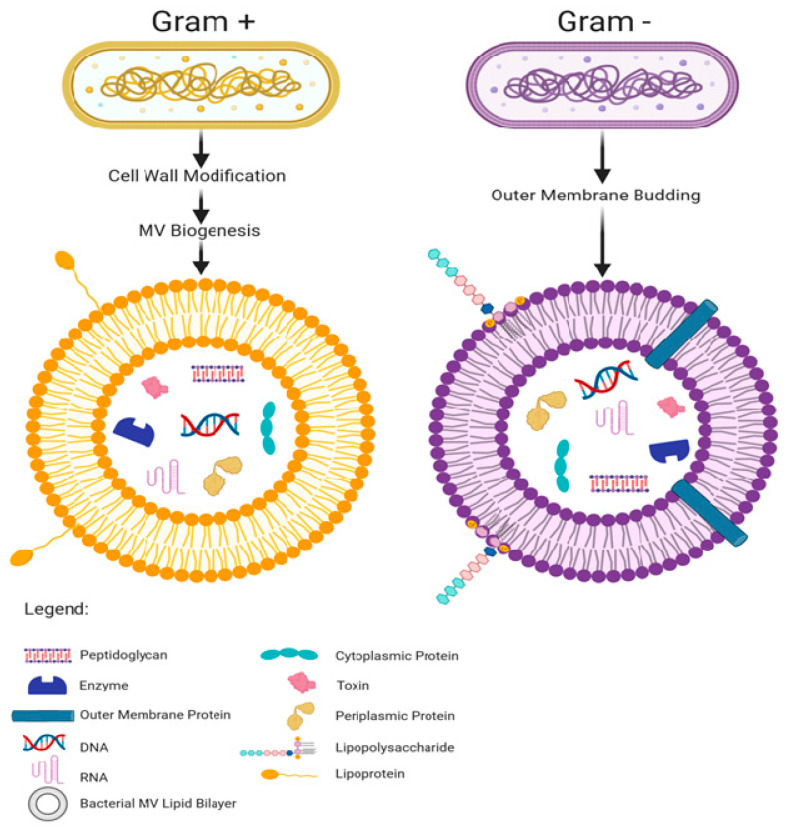
Bacterial membrane vesicle (MV) biogenesis and cargo types, comparing Gram negative and positive bacteria.

Bacterial MV, like eukaryotic extracellular vesicles (EV), carry and stably shelter diverse cargo including peptidoglycan, polysaccharides, proteins, DNAs, RNAs, metabolites, enzymes, and toxins, and express many of the same proteins on their outer membrane as are found on the surface of the bacterium that shed them [58,59,60,61,62,63] (summarized in Figure 1). Although MV are shed constantly by commensal bacteria, they are produced even more abundantly in response to heat stress, suggesting that environmental factors can influence MV production [64,65,66].

MV play a role in cross-talk between bacteria and facilitate this communication partly by sheltering hydrophobic signaling molecules and by allowing the signal to be amplified—as the package can contain many copies of the same signaling molecule and shuttle them over a long distance [52]. A species of marine pathogenic bacteria which benefits from both of these MV features, *Vibrio harveyi* has been shown to utilize outer membrane vesicles to transport a hydrophobic quorum sensing molecule, CAI-1, between cells [67]. Bacteria also use MV as a means of expelling damaging molecular species, misfolded proteins, or even viral particles: increased vesiculation during the lytic phase of the viral infection increases the survival rate of the bacteria [52,64,68]. Bacterial MV also contain enzymes for the breakdown of nutrients: *P. aeruginosa* derived MV possess a pseudomonas quinolone signal (PQS), a small molecule that has an iron chelating and binding activities, and is also involved in quorum sensing between bacteria [69,70]. By scavenging iron in the MV, which then return to the bacterium, vesicular PQS allows *P. aeruginosa* to be viable in nutrient poor environments that it would not otherwise be able to inhabit [71]. Bacterial MV can enable biofilms to form around bacterial colonies: addition of MV to *H. pylori* culture was seen to incite the formation of a biofilm and 52% of the LPS in a *P. aeruginosa* biofilm resides in MV, rather than the actual bacteria [72,73]. *Haemophilus influenzae* derived MV contain DNA which is thought to be transferred to other bacteria, suggesting that MV may also be used to transfer genetic material among a population of bacteria contributing to horizontal gene transfer [74]. MV can also help microbes outcompete other organisms, for example, *Solfolobus solfataricus* discourages the growth of nearby same-genus bacteria via MV packaged toxins [75].

Crucially, bacterial MV are also involved in inter-kingdom communication—passing through cell membranes into eukaryotic cells, and in the case of gut microbes, through the intestinal wall, and into the bloodstream [50,76]. Bacterial MV interactions with the host can include transfer of virulence factors and toxins during infection, gene transfer, antimicrobial protection, and nutrient uptake [77].

Pathogenic bacteria use MV to facilitate the infection and, therefore, their survival, in a number of ways including suppression of the immune system, and delivery of survival-promoting RNA to host cells [78,79].

With regard to commensal organisms, Bryant et al. [58] performed an in silico analysis on the metabolites from MV of a bacterium native to the human gut, *Bacteroides thetaiotaomicron,* and using flux balance analysis (FBA) predicted whether metabolites were intended for the host or other bacteria. The host, in this case a mouse, had to have an enzyme capable of converting the metabolite into a useful product in order for that metabolite to be considered ‘intended’ for the host. The investigators found that *B. thetaiotaomicron* preferentially loads MV with chemicals that the mouse can metabolize, but does not seek to target any particular pathway [58]. MV increased the range of metabolites that the mouse cells take up by about 12% [58].

MV are also thought to travel through the host circulation, able to pass through the tight junction of the intestinal wall—as indirectly evidenced by the presence of bacterial RNA, which could not survive unprotected in the circulation, in the blood of healthy individuals [80,81,82,83]. Once they are in the blood it is implied that they travel throughout the entire body and may deliver cargo to several organs including the brain.

## 2. The Role of Bacterial MV in Microbial Communication to the Brain

### 2.1. Bacterial MV Modulate Immune Activity

There is good evidence linking the peripheral immune system to CNS function. Depression and anxiety have been associated with disruption of immune regulation and an inflammatory immune profile [84,85,86] In animal models, immune activation has been linked to changes in anxiety, memory, social behavior, and learning [87,88,89,90,91], and the development of symptoms associated with mood and cognitive disorders [92,93,94,95].

Deficiencies in mast cells and knockdown of the inflammatory cytokine IL-4 both independently result in increased anxiety-like behavior [89,91]. IL-4 also plays a role in the formation of memories, as does interlferon-γ [87,88]—while interleukin-1 is involved in regulating social behavior [90]. T regulatory cells (Treg), which drive immunosuppressive and anti-inflammatory responses, have been reported at reduced levels in peripheral blood of subjects with major depression [96] and PTSD [97], and have been demonstrated to modulate anxiety- and depressive-like behavior in mice [98]. Furthermore, mouse models also indicate that Treg plays a role in the microbiota–gut–brain axis, being demonstrated to mediate the anxiolytic and antidepressant effects of an *L. rhamnosus* strain [33].

Immune cells at the interface with the external environment, such as dendritic cells and epithelial cells of the gut readily interact with commensal bacteria and their MV [99,100]. Bacterial MV express a concentrated array of microorganism-associated molecular patterns (DNA, RNA, lipoproteins, LPS, and peptidoglycan) that enable engagement with pattern recognition receptors on host immune cells to activate signaling cascades. MV derived from several pathogenic bacteria have been demonstrated to be strong activators of cytoplasmic peptidoglycan sensors NOD1 and NOD2, and Toll-Like receptors (TLR) resulting in activation of necrosis factor (NF)-κB [101,102,103,104]. MV induced activation of this pathway in epithelial, endothelial, and innate immune cells leads to NF-κB translocation to the nucleus, resulting in upregulation of cytokine production as well as adhesion molecule expression, which can, in turn, modulate cell trafficking [101,102,103,104].

Commensal/probiotic derived MV can also influence immune responses. *Akkermansia muciniphila* derived MV can attenuate the production of proinflammatory cytokines in intestinal epithelial cells [105], while MV from *Bacteroides fragilis* were demonstrated to both inhibit proinflammatory cytokines and induce the production anti-inflammatory cytokines in an epithelial cell line [106]. *Bacteroides fragilis* MV also stimulate dendritic cells to induce an immunomodulatory Treg response that can suppress mucosal inflammation in dextran-sulphate sodium (DSS)-induced colitis [107,108]. Similarly, oral treatment with MV from a strain of *Lactobacillus rhamnosus* enhanced expression of immunoregulatory markers, IL-10 and hemeoxygenase-1, in dendritic cells and subsequently induce Tregs in Peyer’s patches and mesenteric lymph nodes of mice [62], effects that had previously been identified for the whole organism [109]. *Bacteroides vulgatus* derived MV have also been demonstrated to induce a tolerogenic phenotype in bone marrow derived dendritic cells [110].

Nissle strain *E. coli* derived MV can lower proinflammatory markers in DSS-induced colitis and upregulate IL-22 in colonic explants [111,112] while MV derived from three species of *Lactobacillus*, *kefir*, *kefiranofaceins*, and *kefirgranum*, all inhibit the production of proinflammatory cytokines in a 2,4,6-trinitrobenzenesulfonic acid (TNBS)-induced IBD mouse model [113]. Vesicles from another *Lactobacillus* species, *sakei*, increase the production of IgA in the intestine [114]. In mouse models, *Bifidobacterium longum* and *Bifidobacterium bifidum* derived vesicles have been found to suppress allergy-related diarrhea via induction of mast cell apoptosis and promote naive T cell development into Tregs, respectively [115,116]. Overall, there is strong evidence that gut bacterial MV can modulate immune responses that have been linked to gut–brain communication opening the possibility that MV interactions with the immune system contribute to the ability of gut bacteria to modify host brain function and behavior.

### 2.2. Bacterial MV Stimulate the Local Nervous System

Published evidence that commensal bacterial MV interact with the peripheral nervous system is limited. However, Al-Nedawi et al. [62] found that exposure of the mouse intestinal lumen to *L. rhamnosus* derived MV increased the excitability of afferent neurons in the myenteric plexus. This may be significant to gut brain signaling as *L. rhamnosus* signals to the vagus nerve via intrinsic primary afferent neurons in the myenteric plexus [17] and the vagus nerve is critical to the effects of the bacteria on brain activity [117] and behavior [3]. Other investigators have also demonstrated that whole gut bacteria stimulate the vagus nerve indirectly via released metabolites interacting with the enteric nervous system [12,118]. That bacterial MV could utilize the vagus nerve to mediate gut–brain communication was suggested by a recent study by Lee et al. [119]. The investigators performed a fecal transplant from both older humans and aged mice into young mice and examined changes in dementia-related behavior and physiology. Lee et al. [119] demonstrated that MV derived from *Paenalcaligenes hominis* caused cognitive deficits in the brain as well as increasing the number of activated microglia in the hippocampus [119]. Cognitive deficits significantly reduced, and the hippocampal cell populations normalized in vagotomized animals suggesting that MV interaction with the vagus nerve is at least partially responsible for the MV induced changes to the brain [119].

### 2.3. Bacterial MV Carry Psychoactive Cargo

Several bacteria and other microorganisms have been shown to produce neurotransmitters including GABA; norepinephrine, serotonin, and dopamine [44,120]. *Lactobacilli* produce a range of neurotransmitters among the various species in its genus—with some single species like *Lactobacillus plantarum* producing multiple neurotransmitters [121]. Zakharzhevskaya et al. [122] analyzed MV isolated from pathogenic and nonpathogenic strains of *Bacteroides fragilis* and found the pathogenic strain contained histamine in addition to the enzyme catalyzing histidine decarboxylation to histamine. Histamine is a neurotransmitter involved in regulation of intestinal function and modulation of local immune responses, in addition to acting centrally to influence brain functions related to sleep and wakefulness, learning and memory, anxiety, locomotion, feeding, and drinking [123]. GABA, the major inhibitory neurotransmitter, and its biosynthesis intermediates α-ketoglutarate and glutamate were also detected in MV produced by *B. fragilis* [122]. These findings suggest that not only do gut bacteria contain brain and behavior altering neurotransmitters, but they have the capacity to package and excrete these molecules in MV, where they can be protected and shuttled through the body at concentrated levels. While altered neurotransmitters levels have been associated with a range of neurological disorders (deficits of serotonin and GABA have been proposed to underlie major depression [124,125] while loss of dopamine production is a defining characteristic of Parkinson’s disease [126]) the potential influence of neurotransmitter-transporting bacterial MV on such conditions has yet to be investigated.

Another hypothesized means through which bacterial vesicles can modulate brain function is through delivery of bacteria derived nucleic acids to the brain [76]. Extracellular RNA (exRNAs) carried by bacterial MV can bind to host RNA-induced silencing complex (RISC), indicating that microbial exRNAs may behave as host gene regulators [127]. Indeed, bacterial exRNA have been demonstrated to downregulate inflammatory cytokine production in epithelial and immune cells [128,129]. It has also been suggested that microbial exRNA may function similarly to eukaryotic Long Non-Coding RNAs (LnRNAs) and act as epigenetic regulators [130]. This is significant as epigenetic modification by histone acetylases and deacetylases modulates gene expression involved in synaptic plasticity related to brain development, learning, and memory [131,132].

The presence of RNA and nucleic acids are much stronger evidence of MV delivery than protein or small molecules from bacteria, because RNA is a lot less stable and unlikely to survive the voyage through the circulation without being sheltered. Emery et al. [133] assessed bacterial RNA in the brain of Alzheimer’s patients, postmortem, compared to subjects without Alzheimer’s. The bacterial RNA most prevalent in brain from Alzheimer’s patients was found to be derived from families including: *Propionibacteriaceae*, *Staphylococcaceae*, *Corynebacteriaceae*; as well as more generally from the phyla Proteobacteria and Firmicutes [133]. As a fraction of the total bacterial RNA reads, Actinobacteria and Firmicutes were more prevalent in Alzheimer’s disease brains than normal brains, and Proteobacteria and *Bacteroides* were more prevalent in normal brains than diseased [133]. It is recognized that most bacteria associated with Alzheimer’s disease originate in the oral microbiome and several correlations have been drawn between poor oral hygiene and dementia [134,135].

Zhan et al. [136] similarly found pilli protein from *E. coli* K99 and lipopolysaccharride (LPS) in the brain tissue of patients with Alzheimer’s disease. LPS was present in both diseased and normal brains but in the grey matter of diseased brains LPS appeared in amyloid plaques in the cortex [136]. Likewise, the bacterial pilli protein was observed in neuron-like cells in the cortex of Alzheimer’s disease patients but the same could not be said for normal brains in which ependymal cells hosted the protein [136]. The glutamate decarboxylase B transcript expression was detected in both diseased and healthy brains and in database search was found to be identical to those known in 115 *E. coli* strains and five *Shigella* strains [136].

It must be noted that another possible explanation for these findings is the direct colonization of the brain by these bacteria for which there is some evidence [137,138]. However, the possibility that bacteria can directly modulate brain function without colonization should be considered and numerous studies indicate that MV derived from pathogenic bacteria do perturb, and even cross, the blood–brain barrier [78,139].

*Aggregatibacter actinomycetemcomitans*, a Gram negative, oral, pathogenic bacteria that causes periodontitis (and may also be implicated in Alzheimer’s disease) produces MV that contain several RNAs thought to be important to its pathogenesis [78]. Han et al. [78] showed via two-dimensional light sheet microscopy that *A. actinomycetemcomitans* derived MV cross the blood–brain barrier, appear in brain vessels 4 h following cardiac injection, and 24 h after cardiac injection they appear in the cortex region. The MV were also shown to be responsible for an increase in TNF-α production likely due to the delivery of extracellular RNA altering Toll-like receptor 8 and NF-kB signaling pathways [78].

Wispelwey et al. [139] found that vesicles derived from *Himophilus influenzae* type b increased blood–brain barrier permeability significantly [139,140]. The permeability increase induced by MV was found to be close to that caused by LPS [138].

### 2.4. Bacterial MV are Capable of Altering Behavior and Gene Expression in the Brain

Lee et al. [119] found that oral gavage of MV derived from *Paenalcaligenes hominis*—a bacterium found 4.3 times more abundantly in the guts of aged mice—resulted in an increase of bacterial 16S rDNA associated with impaired cognitive function, an increase of activated microglia, and an increase in dementia-related brain inflammation [119]. These MV also increased dementia-like behavior measured as less time spent spontaneously alternating arms of a Y-maze [119]. That vagotomy prevented many of these behavioral and brain changes suggests that either MV interact with the vagus nerve which stimulates changes in the brain, or travel through the vagus nerve to reach the brain—which might better explain the presence of bacterial 16S rDNA [119]. Intact MV were not however, detected in the hippocampus regardless of vagus nerve integrity [119]. MV also increased blood LPS, suggesting that they were also present in the circulation, regardless of vagal nerve integrity.

In in vitro studies, Choi et al. [141] identified that MV from a Gram positive probiotic native to the gut, *Lactobacillus plantarum,* upregulated expression of BDNF transcripts as well as proBDNF protein in HT22 hippocampal cells after the induction of depression-associated changes by glucocorticoid (GC) treatment. Sirtuin 1, a deacetylase that contributes to cellular regulation in response to stress, was identified as a potential mediator of the MV induced rescue of BDNF expression following GC treatment in HT22 cells, a finding which was confirmed in vivo using mice treated with restraint stress to generate a depressive phenotype [141]. MV injected intraperitoneally either during restraint stress, immediately following restraint stress or 2 weeks following stress exposure normalized BDNF expression and stress induced behaviors [141] to a similar level as the SSRI, imipramine, strongly suggesting antidepressant like action of the MV [141].

Taken together, evidence suggests that the MV shed by both Gram positive and negative bacteria may play a significant role in brain health, mood disorders, and the behaviors they inform.

## 3. Eukaryotic Intestinal Cell Extracellular Vesicle Signaling in Response to Contact with Microbiota

While not the focus of the current review it should be noted that another possible role for vesicular signaling in microbe to brain communication is that gut cells of the host produce their own vesicles in response to contact with specific commensal bacteria and that these vesicles communicate with the brain.

Mammalian cells can release distinct types of extracellular vesicle (EV), including exosomes, microvesicles, and apoptotic bodies; a classification based on intracellular origin [142]. Mammalian EV range from 30 to 1000 nm in size and contain a variety of cargos, including coding and noncoding RNAs (e.g., mRNA, miRNA, lncRNA), proteins, and lipids. Cells can package a distinct set of biomolecules into EV via endogenous sorting mechanisms, and EV are released constitutively or after stimulation. EV from mammalian cells facilitate local and long-distance signaling, being internalized by recipient cells either by fusion with the plasma membrane or via endocytosis. Once delivered, the mammalian EV cargo can alter the phenotype of recipient cells and in doing so regulate a variety of physiological responses [142].

Host derived EV have been identified as potentially contributing to several neurodegenerative conditions. Amyloid β protein (Aβ), which aggregates in Alzheimer’s disease (AD), is found in EV isolated from the plasma and brain of AD patients. These EV can spread toxic Aβ oligomers and cause toxicity in neurons [143]. It is also suggested that exosomes secreted by both activated microglia and neurons play an important role in Parkinson’s disease; exacerbating disease severity and progression through α-synuclein spreading and increased neuroinflammation [144]. Exosomes isolated from the brain of patients with Amyotrophic lateral sclerosis (ALS) contain TAR DNA-binding protein 43 (TDP-43), a major pathological protein in the disorder [145]. Evidence that these EV may have a role in disease progression comes from the observation that they have the potential to propagate TDP-43 pathology to healthy cells [145]. Thus, there is increasing evidence suggesting that eukaryotic EV carry pathological molecules and thereby contribute to the progression of neurodegenerative diseases. Conversely, there is evidence that EV derived from specific host cells, particularly mesenchymal stem cells (MSC), can be protective against neurodegenerative disorders. EV secreted from human adipose tissue derived-MSC are enriched in multiple proteins possessing neuroprotective and neurogenic activities [146]. These MSC derived EV were demonstrated to enter the brain following intranasal administration in mice and ameliorated neurologic damage caused by glutamate toxicity [146]. Intranasal administration of the EV also increased neurogenesis and rescued memory deficits in a mouse model of AD [146]. Similarly, intranasal administration of MSC-derived EV improved motor function in a rat model of PD [147].

The quantity and content of EV produced by intestinal epithelial cells has been demonstrated to be alerted following pathogen infection [148] or injury [149]. It is likely that the gut microbiota composition and/or exposure to specific commensal or probiotic organisms also influences EV production by intestinal epithelial cells. Commensal bacteria have been shown to influence neurometabolite levels in intestinal cells [150]. Exposure to Nissle strain *E. coli* was shown to upregulate 5-HT production, as well as 5-HTP—an intermediate that is later converted to 5-HT, in human enterochromaffin cells [151]. Once synthesized, 5-HT is stored by enterochromaffin in large dense core vesicles which can be released into the blood [152,153]. Shed eukaryotic EV can also cross the blood–brain barrier, implying that these host created extracellular vesicles could be another mechanism by which vesicles mediate the gut–brain crosstalk [154,155,156].

## 4. Conclusions

**Figure 2:**
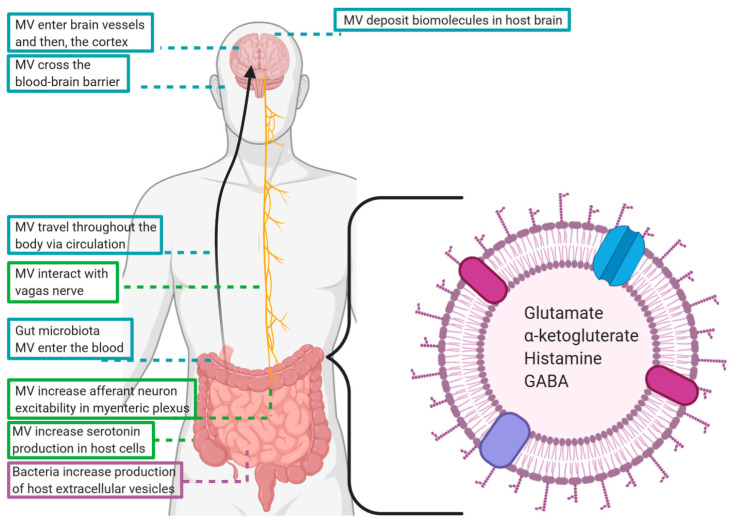
Paths taken by commensal microbiota derived membrane vesicles (MV) and the ways in which they may influence cognition and behavior.

There is a growing body of evidence suggesting that the membrane vesicles of commensal bacteria are integral components of the microbiota–gut–brain axis. At the level of the intestine, MV engage in many of the same local communicative activities as parent bacteria, but additionally have greater access to the circulation than whole microbes and are capable of travelling to the CNS. This way, MV facilitate delivery of concentrated signaling molecules and delicate cargo, such as RNA, that could not survive the journey from gut to brain unprotected (summarized in Figure 2).

This is a nascent field of research in which many questions remain to be addressed; with even basic aspects of the interaction between MV and cells of the immune, and peripheral and central nervous systems poorly understood.

Eukaryotic extracellular vesicles are known to have natural affinity towards certain tissue types and organs in a complex manner that relies on their integrin profile, tetraspannin profile, fibronectin expression, lipid composition, glycan composition, and charge [157,158,159,160,161,162,163]. Future research should address the degree to which bacterial MV are targeted to specific host cells and whether some of these same mechanisms are involved in such targeting. Furthermore, identifying how the microenvironment and ecology of the microbiota influence both the production and content of MV will help us understand the role played by this signaling process within the microbiota–gut–brain axis.

Given that, in many cases, parent bacteria and MV are shown to elicit the same biological responses it will be important to determine the relative contribution of the whole organism versus microbe derived vesicles in gut–brain signaling under physiological conditions in vivo. This question has been addressed in relation to mammalian intercellular signaling through the use of inhibitors of vesicle production and release [164]. A better understanding of cellular machinery involved in the generation of bacterial MV may allow for the development of similar approaches to explore microbe-host communication.

In addition, also worthy of exploration is the fate of bacterial MV once they reach the brain, how they release their cargo upon entry, what cells they act upon and why, they appear not to be present after delivering their cargo as to date, intact bacterial MV have not been detected in the brain.

To date, the exploration of mechanisms underlying microbial influence on brain and behavior has largely overlooked a communication system that has been conserved through all domains of life and allows bacteria to directly regulate biological responses of the host without the need for cell–cell contact. Advances in our understanding of bacterial MV as an inter-kingdom signaling system will almost certainly provide valuable insights to the microbiota–gut–brain axis and potentially lead to more effective approaches to psychobiotic therapy. Currently, we are not aware of any published studies directly assessing the therapeutic potential of MV derived from commensal or probiotic bacteria in neurological disorders, however, it is clear such studies are warranted and, with increasing recognition of this form of inter-kingdom signaling, are likely to emerge in the near future.

## Abbreviations

DOAJ	Directory of open access journals
TLA	Three letter acronym
LD	linear dichroism
